# Extra-articular Deformity Correction via Clamshell Osteotomy of the Tibia With Simultaneous Bilateral Total Knee Arthroplasty

**DOI:** 10.7759/cureus.61186

**Published:** 2024-05-27

**Authors:** Mansour A AlSaflan, Abdullatif A AlRajeh, Abdullah M AlHossan

**Affiliations:** 1 Orthopedic Surgery, King Abdulaziz Hospital, AlAhsa, SAU; 2 Orthopedic Surgery, King Fahad Military Medical Complex, Dhahran, SAU; 3 College of Medicine, Alfaisal University, Riyadh, SAU

**Keywords:** bilateral total knee arthroplasty, clamshell osteotomy, shaft femur, tibial osteotomy, total knee arthroplasty technique

## Abstract

Total knee arthroplasty (TKA) aims to alleviate severe pain and functional impairment in advanced knee arthritis. However, extra-articular deformities must be addressed preoperatively to ensure optimal implant positioning and soft tissue balancing. We present a case of a 58-year-old man with a history of a right tibial malunion and left femoral shaft fracture, who developed progressive bilateral knee osteoarthritis. He underwent simultaneous bilateral TKA with a right tibial clamshell osteotomy to correct a 17° varus deformity and left femoral hardware removal to accommodate a long stem implant. At one year follow-up, the patient had satisfactory functional outcomes with restoration of mechanical alignment and bony union at the osteotomy site. This case demonstrates the utility of the clamshell osteotomy in correcting tibial malunion to facilitate TKA with long stems, reducing the degree of intra-articular correction required.

## Introduction

Total knee arthroplasty (TKA) aims to relieve severe and deteriorated knee pain and impairment. However, before a total knee replacement, significant abnormalities in the lower extremities, especially in the diaphysis area, must be corrected. It facilitates the ligament's release and helps to reduce the quantity of bone that will be sacrificed. The standard of care for end-stage osteoarthritis in the knee is TKA [1-2]. Twenty percent or more of patients whose osteoarthritis is severe enough to require TKA have bilateral involvement, meaning they require bilateral TKA (BTKA) [3]. The advent of BTKA has heralded a new era in joint replacement surgery, promoting efficiency and patient satisfaction through simultaneous procedures that ensure a single recovery timeline, thus significantly reducing the patient's time away from daily activities and occupational responsibilities [4].

For an arthroplasty surgeon, the challenge is further compounded when confronting diaphyseal malunion, which can disrupt normal gait kinetics, compromise aesthetic appearance, and escalate the risk of osteoarthritis [5]. Furthermore, the management of long bone fractures linked to diaphyseal malunion presents an extra level of difficulty [6]. Without an osteotomy or multiplanar plate contouring, it is practically impossible to do intramedullary nailing or plating for a long bone fracture with a pre-existing diaphyseal malunion. It was designed to be managed using clamshell osteotomy. Russell and Graves initially reported in 2009 that it stabilizes an associated fracture, if it exists, and permits the diaphyseal malunion to be bypassed to restore the coronal and sagittal plane anatomical axes of the long bone using an intramedullary nail [7,8].

A case of TKA following correction of extra-articular femoral deformity resulting from a malunited femoral shaft fracture. As far as we know, this is the first instance of a total knee replacement following clamshell osteotomy-assisted correction of an extra-articular abnormality in the tibia [9,10]. A case series of 10 patients (4 femoral and 6 tibial) with posttraumatic diaphyseal malunions was presented by Russell and Graves. All patients displayed complete correction of translation, rotation, and joint-line orientation angles, as well as correction to within 4° in the coronal and sagittal planes and 2 cm in the limb-length discrepancy [9]. When there is an extra-articular deformity, alignment can be restored through intraarticular correction using soft tissue balancing and prosthesis placement, osteotomy at the deformity location, or osteotomy away from the deformity [11].

We present the case of a patient with a history of road traffic accident 37 years ago, who got right tibia and left femur shaft fractures and their respective treatment; conservative for the tibia and an open reduction internal fixation via plates and screws for the femur in another hospital and the surgical techniques followed by bilateral knee arthroplasty utilizing long stems.

## Case presentation

Case history

**Figure 1:**
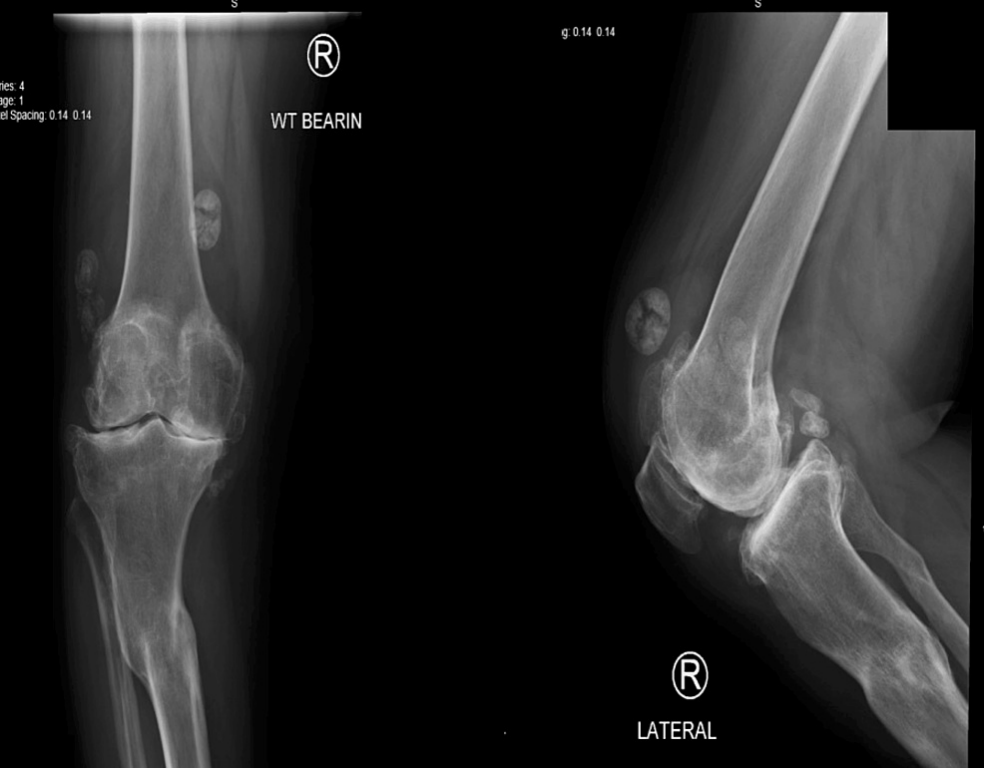
Radiograph showing tibial varus deformity

**Figure 2:**
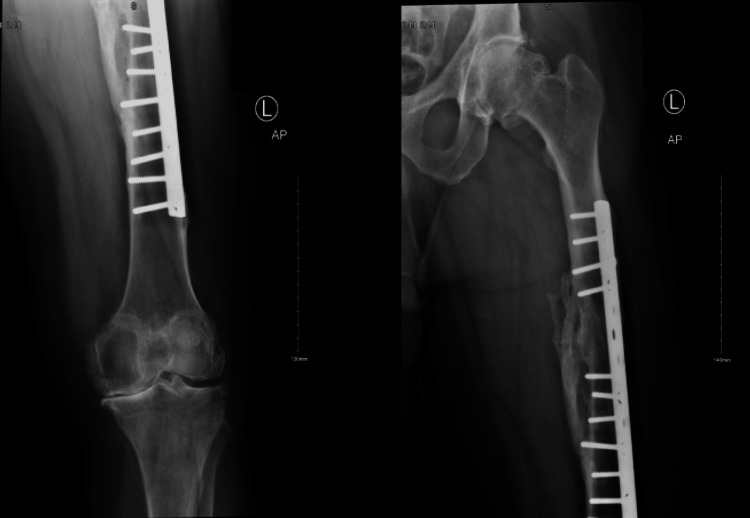
Radiographs showing AP view for left femur fracture plate fixation

**Figure 3:**
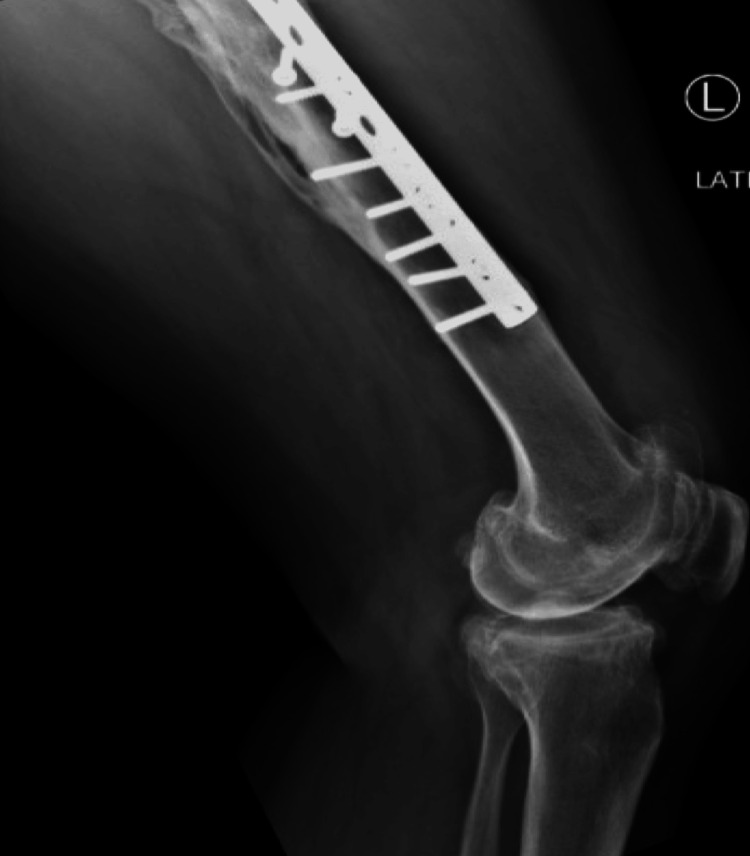
Lateral view for left femur fracture plate fixation

**Figure 4:**
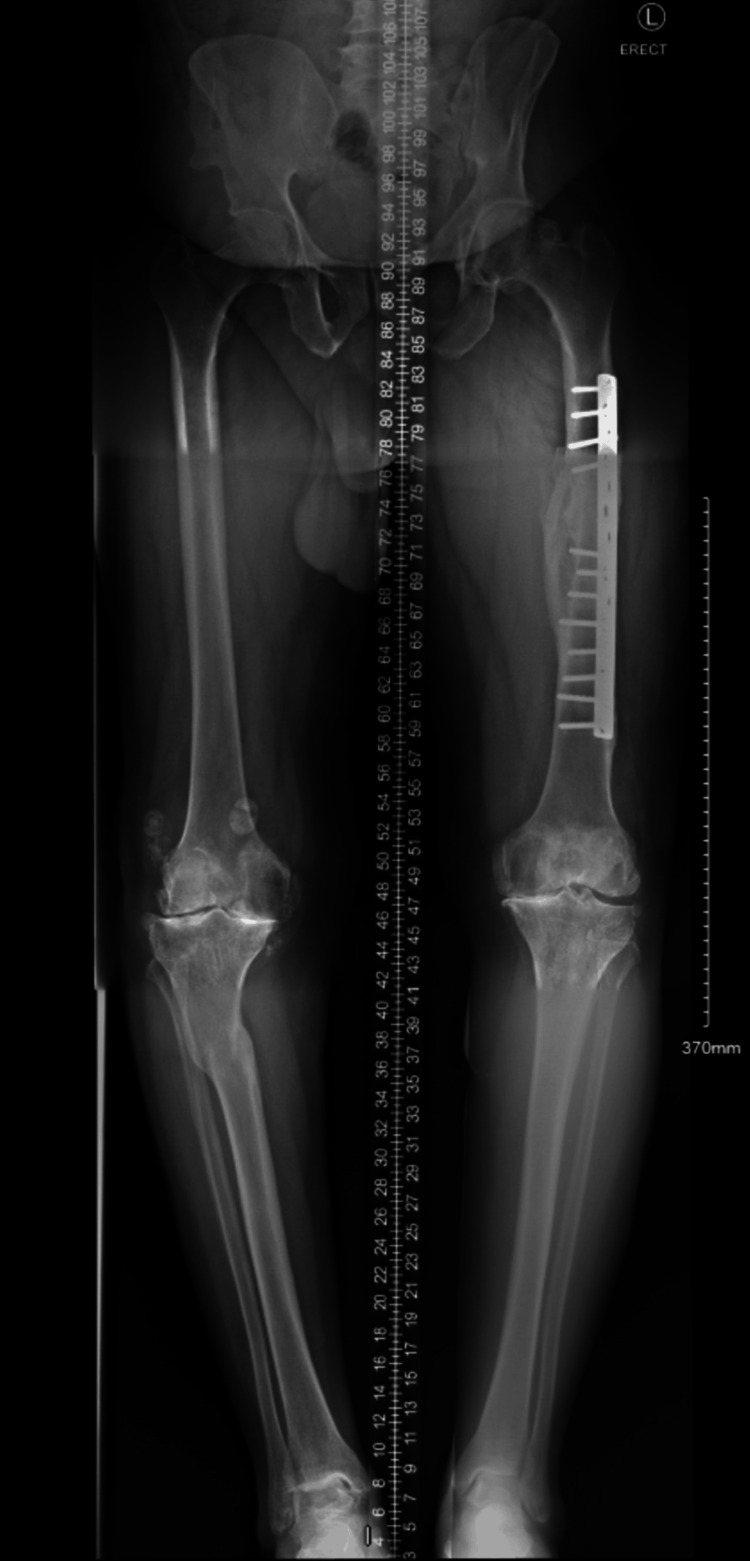
Full-length standing radiography showing right-sided post-traumatic tibia varus deformity of 17°

We present a case of a 58-year-old Saudi male with a history of road traffic accident 37 years prior, resulting in right tibial and left femoral shaft fractures presented with progressive bilateral knee arthritis, as shown in (Figures 1, 2, 3). His tibia was treated conservatively at the time, in which, he developed post-traumatic tibia varus deformity as a consequence, as shown in Figure 4. His comorbidities included diabetes and hypertension. He used non-steroidal anti-inflammatory drugs (NSAIDs) and a cane for ambulation assistance but had activity limitations and difficulty climbing the stairs due to his advanced osteoarthritis. Upon examination, he walked with an antalgic gait. The active range of motion on the right measured 5°-90°of flexion. He exhibited joint line tenderness medially and pseudolaxity with varus stressing compared to the contralateral side.

Radiographs and long leg films showed tri-compartmental degenerative changes with 17° varus alignment of the right tibia due to remote fracture malunion. Advanced patellofemoral arthritis was also observed. CT confirmed extra-articular tibia deformity without intra-articular subluxation or osteonecrosis.

Surgical technique

**Figure 5:**
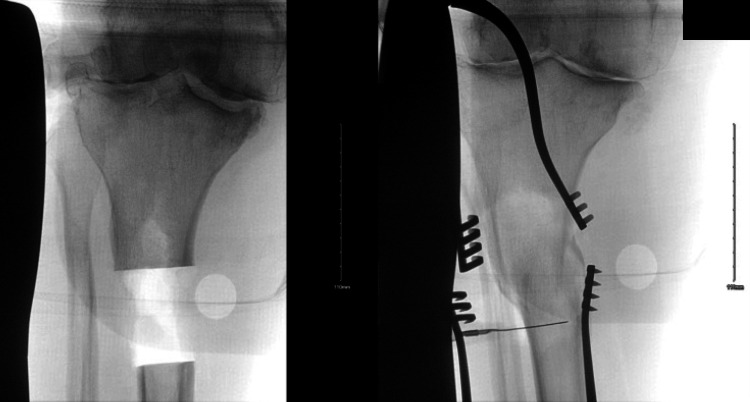
Intraoperative fluoroscopic image showing osteotomy level being created in a diaphyseal segment

**Figure 6:**
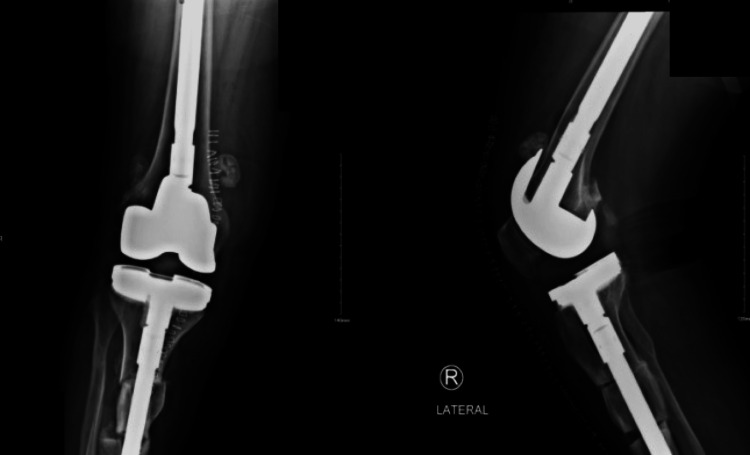
Right side anterior-posterior and lateral view radiograph for total knee arthroplasty

**Figure 7:**
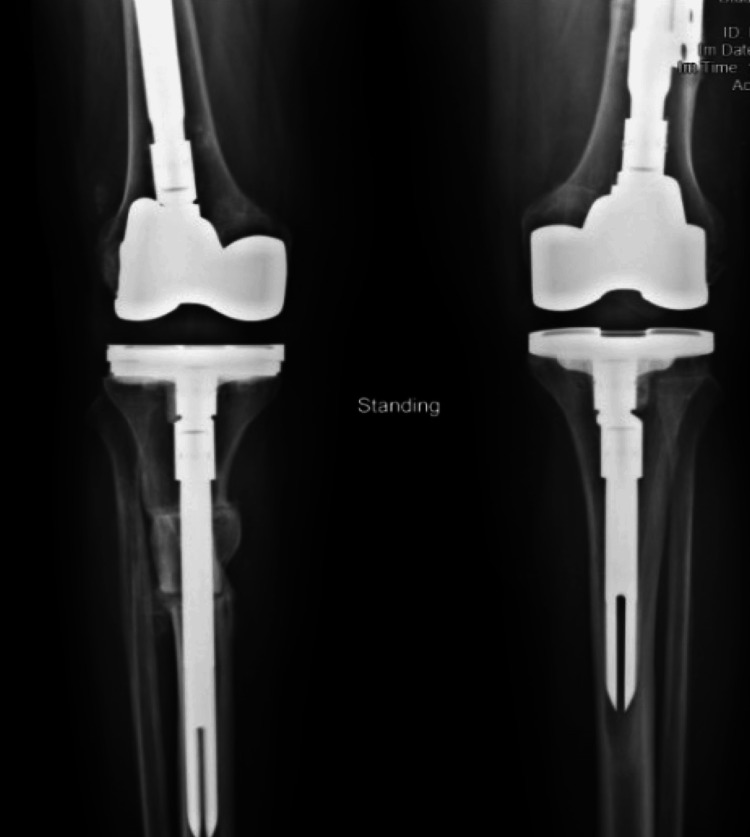
Three months post-operative standing position of total knee arthroplasty

**Figure 8:**
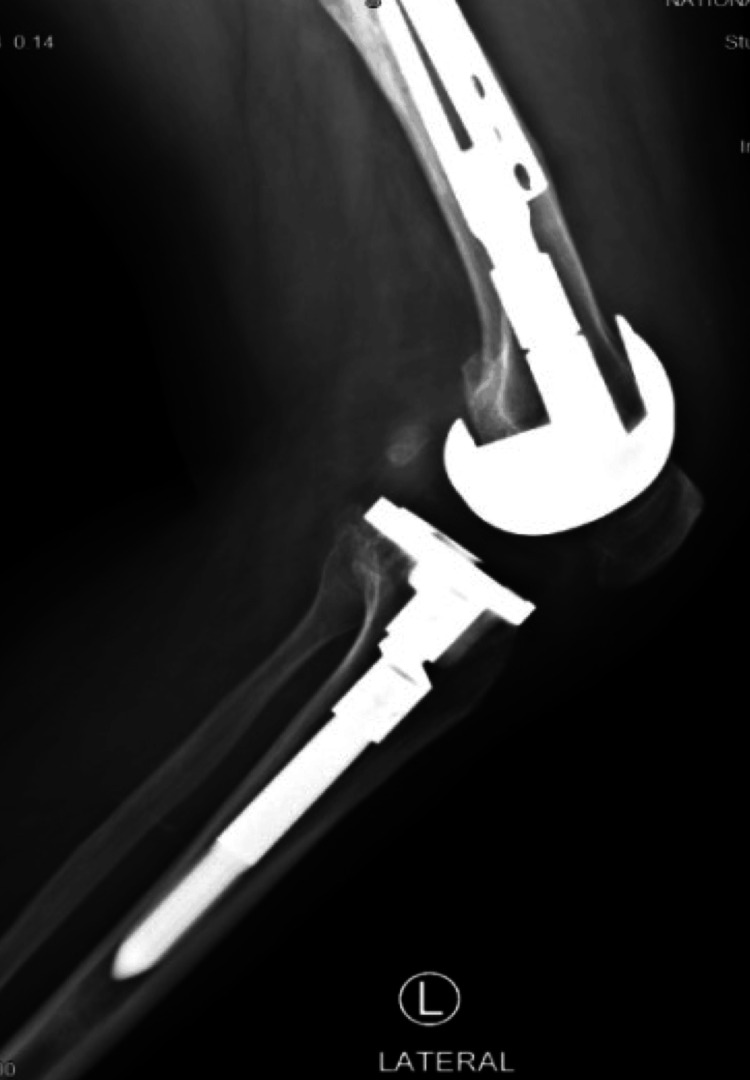
Three months post-operative lateral view taken after left side total knee arthroplasty

**Figure 9:**
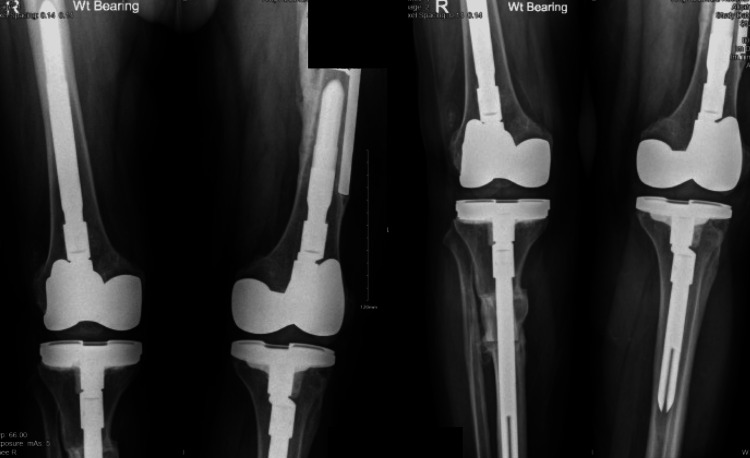
Radiograph showing follow-up one-year mark

**Figure 10:**
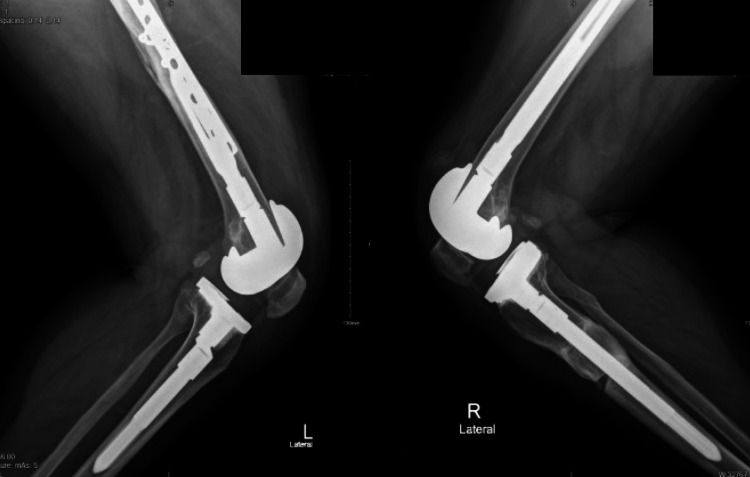
Left and right sides, lateral view after one-year mark follow-up

**Figure 11:**
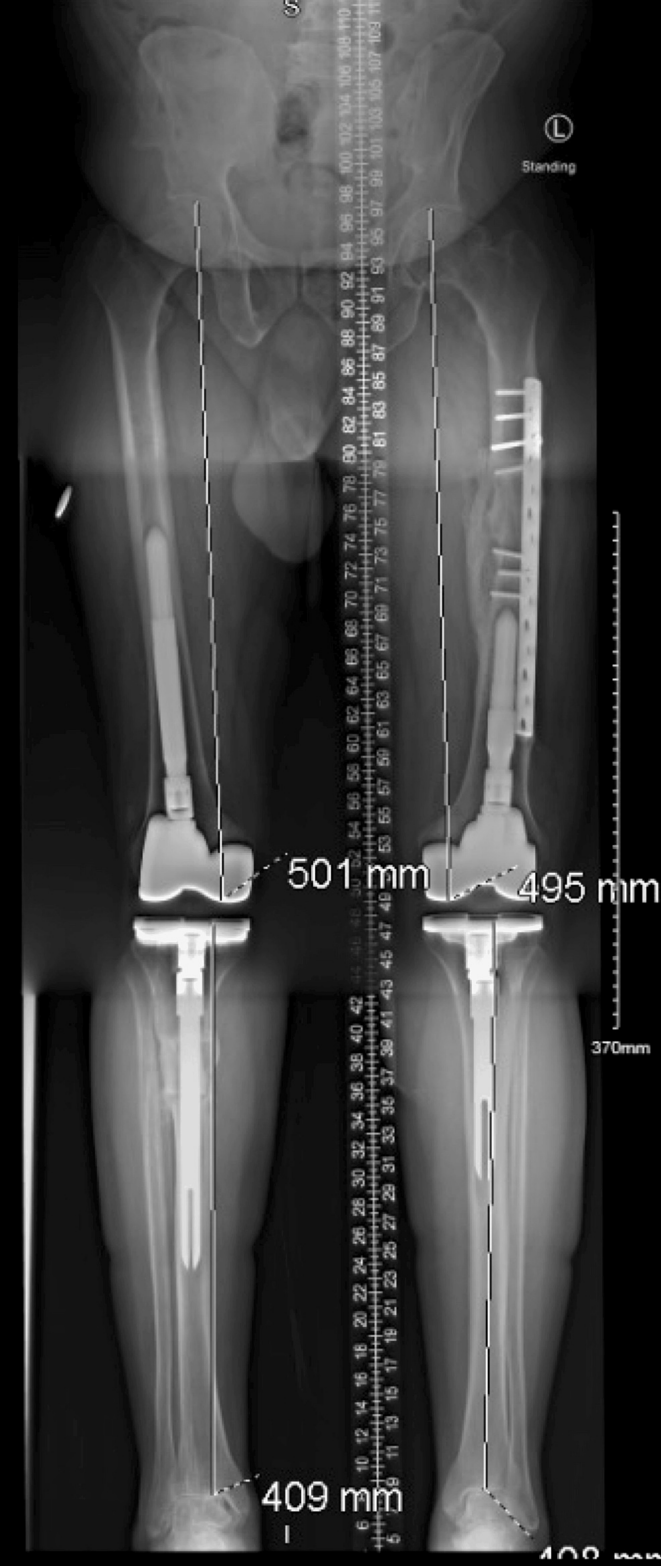
Full-length standing radiography showing mechanical restoration after total knee arthroplasty

The patient was indicated for bilateral TKA, correction of the tibia varus (17°) was needed. A two-stage approach was planned. (1) Right tibial clamshell osteotomy with long stem TKA; (2) removal of left femoral hardware doing a TKA. For the tibial procedure, the limb was prepared and draped free on the operating table, through a midline knee incision with extension distal to the deformity. After localizing the osteotomy sites fluoroscopically, drill holes created a plane of stress risers along the intended clamshell path, beginning posterior to the anterolateral subcutaneous ridge and oriented posteromedially. An oscillating saw made transverse osteotomies proximally and distally, while osteotomes separated the longitudinal osteotomy in the sagittal plane of the clamshell. The osteotomized tibial segment was opened like a clamshell using laminar spreaders until the medullary canal was reconstituted. A ball-tipped guide wire was passed across the clamshell osteotomy and reaming over the wire allowed progressive correction of the deformity under the image intensifier as the anatomic axis template (Figures 5, 6). The osteotomy gaps were filled with the clamshell osteotomized bone after recontouring the edges. After proximal tibial resection and implantation of a long stem tibia, a standard TKA implant was inserted while respecting physiologic joint line height and soft tissue tension. A tibial rod slightly smaller than the last reamer used is then inserted, and the rod is locked in place at the top. The positioning pillow is removed, and the leg is laid flat for final adjustments to length and rotation, using the other leg as a reference. Adjustments are made with manual traction, under image intensifier guidance. The front compartment is then gently pulled back to inspect the osteotomy areas, which is filled with the bone osteotomized bone and from the reaming substitutes. The outer muscle layer is sutured back, leaving some space in case of swelling to prevent compartment syndrome. The left femoral procedure involved the removal of the most distal five screws via a lateral approach to accommodate the space of the long stem. Standard primary TKA midline parapatellar approach was carried with appropriate soft tissue releases to balance the knee in coronal and sagittal planes. Post-surgery care patients are hospitalized and given control over their pain management through patient-controlled analgesia (PCA) and oral painkillers, with vigilant monitoring for compartment syndrome, hence avoiding regional anesthesia. Antibiotics are administered intravenously for 24 hours following the operation. Mobilization is encouraged from day one post-op under a therapist's guidance, initially with crutches and toe touch weight-bearing on the right lower limb, gradually increasing to full weight-bearing by the 12-week mark. Thrombosis prevention is managed with anticoagulants until discharge. Outpatient follow-ups with periodic radiographs at three months (Figures 7, 8) and at one-year follow-up (Figures 9, 10, 11), confirmed maintenance of alignment without complications. By one-year of follow-up, the patient had achieved bony union at all osteotomy sites with well-fixed components and restoration of mechanical alignment.

## Discussion

TKA has great results and a low rate of complications in several case studies. However, in certain circumstances, such as those involving pre-existing bone deformity or soft tissue pathology, the rate of complications might increase from 5% to 41% [12]. While sagittal plane alignment is seldom discussed, much research describes coronal plane alignment in anatomical, mechanical, and vertical axes [13]. The line connecting the lateral epicondyle to the anterior boundary of the greater trochanter is known as the palpable sagittal axis, and it is 2.4° flexed in an observational study of 76 TKA by Seo et al. [14]. The coronal plane is given a lot of weight when restoring the mechanical axis; however, the sagittal plane's mechanical axis is given the least weight in the literature. According to a study by Wang and Chen, intraarticular correction is possible for the femur at 15° recurvatum and 16° procurvatum [15]. This is due to the fact that the knee is a hinge joint that only moves in the sagittal plane, making deviations readily hidden. When there is flexion deformity rather than recurvatum, it is more crucial to restore the sagittal mechanical axis [11]. With 15 knees, Madelaine and Villa [16] reported in 2014 that the mean mechanical axis (MA) was corrected from 19.2° preoperatively to 10° postoperatively. At a 22-month follow-up, the study's overall survival rate was 86.7%.

In the context of a distant tibia fracture, our patient showed a 17° varus deformity that was treated conservatively. The resulting malunion of the tibia led to asymmetric wear and degeneration of the knee. In a retrospective analysis of 15 patients with extra-articular deformity, Wang and Wang discovered that patients with an average coronal plane deformity in the tibia and femur of 20° were successfully treated with intraarticular excision without osteotomy. Within 1° of varus, the mechanical axis was improved [17].

The impact of extra-articular deformities varies based on the anatomic plane involved. As described by Xiao-Gang et al., varus/valgus deformity in the coronal plane alters anatomic landmarks and may necessitate asymmetric bone resection, leading to secondary instability if uncorrected [18]. Sagittal plane deformity requires careful positioning of implants to ensure a neutral joint line. Rotational deformity greater than 10° remains challenging to quantify and correct without osteotomy [18].

A systematic approach based on full-length standing radiographs and cross-sectional imaging when indicated allows quantification of deformities and preoperative planning for intra-articular or extra-articular correction. There are many types of osteotomies and different fixation techniques. While there are proponents of opening wedge osteotomy, in the context of simultaneous TKA, closing wedge osteotomy is intrinsically more stable. Furthermore, it is thought that employing a long-splined stem has several advantages over alternative fixation methods like staples or plating systems. These advantages include the potential for early weight bearing, rotational control, and ease of application. While it is technically demanding to perform a simultaneous osteotomy during TKA, it may be advantageous to address the deformity in a single stage. The patient may face lower risk by avoiding two separate anesthetics [19]. The application of the clamshell osteotomy technique in the acute fracture setting was demonstrated by Pires et al. [6]. Following clamshell osteotomy, nine patients returned to their pre-injury functional status comparable [6,7]. This method enables the simultaneous correction of rotational, translational, and angular deformities away from the knee joint through an intramedullary approach [20]. As compared to other osteotomy techniques requiring plates or external fixators, the clamshell approach provides a biological advantage by preserving an osseous sleeve for healing while using the intramedullary implant to re-establish anatomic alignment [20]. Originally described for complex diaphyseal malunions, this technique can also be applied preoperatively to facilitate TKA in select patients with extra-articular deformities [8].

## Conclusions

In summary, managing osteoarthritis of the knee with significant extra-articular deformity remains one of the most challenging clinical scenarios for orthopedic surgeons. These deformities are uncommon and usually result from fracture malunion. The decreasing incidence of severe deformities in recent decades can be attributed to advances in orthopedic trauma care. As compared to previously described techniques, TKA following clamshell osteotomy provides simpler surgical execution and the benefits of early mobilization and effective deformity correction without the drawbacks of external fixation. Further research is needed to refine indications and document long-term outcomes after this combined procedure.
